# Identification of Common and Subtype-Specific Mutated Sub-Pathways for a Cancer

**DOI:** 10.3389/fgene.2019.01228

**Published:** 2019-11-28

**Authors:** Haidan Yan, Xusheng Deng, Haifeng Chen, Jun Cheng, Jun He, Qingzhou Guan, Meifeng Li, Jiajing Xie, Jie Xia, Yunyan Gu, Zheng Guo

**Affiliations:** ^1^Key Laboratory of Ministry of Education for Gastrointestinal Cancer, Department of Bioinformatics, The School of Basic Medical Sciences, Fujian Medical University, Fuzhou, China; ^2^Key Laboratory of Medical Bioinformatics, Fujian Medical University, Fuzhou, China; ^3^Department of General Surgery, Fuzhou Second Hospital Affiliated to Xiamen University, Xiamen, China; ^4^Department of Systems Biology, College of Bioinformatics Science and Technology, Harbin Medical University, Harbin, China

**Keywords:** mutation, common sub-pathways, subtype-specific sub-pathways, cancer genes, drug targets

## Abstract

The heterogeneity of cancer is a big obstacle for cancer diagnosis and treatment. Prioritizing combinations of driver genes that mutate in most patients of a specific cancer or a subtype of this cancer is a promising way to tackle this problem. Here, we developed an empirical algorithm, named PathMG, to identify common and subtype-specific mutated sub-pathways for a cancer. By analyzing mutation data of 408 samples (Lung-data1) for lung cancer, three sub-pathways each covering at least 90% of samples were identified as the common sub-pathways of lung cancer. These sub-pathways were enriched with mutated cancer genes and drug targets and were validated in two independent datasets (Lung-data2 and Lung-data3). Especially, applying PathMG to analyze two major subtypes of lung cancer, lung adenocarcinoma (LUAD) and lung squamous cell carcinoma (LSCC), we identified 13 subtype-specific sub-pathways with at least 0.25 mutation frequency difference between LUAD and LSCC samples in Lung-data1, and 12 of the 13 sub-pathways were reproducible in Lung-data2 and Lung-data3. Similar analyses were done for colorectal cancer. Together, PathMG provides us a novel tool to identify potential common and subtype-specific sub-pathways for a cancer, which can provide candidates for cancer diagnoses and sub-pathway targeted treatments.

## Introduction

Thousands of mutations are detected for a cancer with the advances of DNA sequencing technologies. The mutation frequencies of most genes are very low (<5%) in all patients of a cancer (Ciriello et al., 2013; Kandoth et al., 2013; Tamborero et al., 2013). Therefore, many algorithms have been developed to identify a panel of genes or pathways that mutate in a significantly high fraction of patients in a particular type of cancer. These identified mutation genes or pathways might be drivers contributing to cancer (Youn and Simon, 2011; Dees et al., 2012; Hua et al., 2013; Merid et al., 2014; Leiserson et al., 2015) or potential diagnosis biomarkers for a cancer (Ece Solmaz et al., 2015; Clifford et al., 2016; Li et al., 2016; Sato et al., 2016). For example, Clifford et al. identified a panel of 400 mutations covering more than 80% of the lung adenocarcinoma (LUAD) patients from The Cancer Genome Atlas (TCGA) database (Clifford et al., 2016). However, in an independent validation dataset, this panel of mutations only covered 55% of 183 patients (Clifford et al., 2016). It is not surprising that the coverage drops so much in the validation dataset because the distribution of somatic mutations is highly heterogeneous (N. Cancer Genome Atlas Research, 2011; N. Cancer Genome Atlas, 2012; Hofree et al., 2013). Thus, the panel of mutated genes, identified only by the mutation information of individual genes, may vary across different independent datasets.

It has been reported that certain pathways are frequently altered across patients of a cancer by mutations in different genes of the pathways (N. Cancer Genome Atlas Research, 2008; Gu et al., 2011; N. Cancer Genome Atlas, 2012). Therefore, a combination of the individual mutations within a pathway (Vaske et al., 2010; Bertrand et al., 2015; Cho et al., 2016; Hristov and Singh, 2017; Shrestha et al., 2017) or a molecular network (Hristov and Singh, 2017) is a preferred method to deal with inter-tumor heterogeneity. To obtain a small subset genes that was more relevant to disease, many methods have been developed to identify sub-pathways or sub-networks. For sub-pathway analysis, most methods are based on the enrichment analysis of differentially expressed genes, such as topology enrichment analysis framework (Judeh et al., 2013), pathway and transcriptome information (Nam et al., 2014), and Subpathway-GM (Li et al., 2013). The methods to extract sub-networks mainly combined mutations with copy number variations to identify modules related with diseases, such as HotNet (Leiserson et al., 2015) and MEMo (Ciriello et al., 2012). Panels of mutation genes have been reported to be a promising way to diagnose a specific cancer (Ece Solmaz et al., 2015; Clifford et al., 2016; Li et al., 2016; Sato et al., 2016). It would be of great significance if we could find sub-pathways mutated in almost all patients of a cancer. Here, we think that the panel of mutation genes within a common sub-pathway will be a reliable diagnostic marker for a cancer when the common mutated sub-pathway is reproducible in different independent datasets of this cancer. However, current methods didn’t consider this application of sub-pathways. On the other hand, a cancer may have different subtypes with different causes and clinical outcomes. Thus, it is also important to obtain subtype-specific biomarkers to guide subtype diagnoses and treatments.

In this study, we developed an empirical algorithm, called PathMG, to identify common and subtype-specific mutated sub-pathways for a cancer. By analyzing multiple mutation profiles of lung cancer, three reproducible common mutated sub-pathways were identified. PathMG was also used to identify LUAD-specific and lung squamous cell carcinoma (LSCC)-specific sub-pathways, respectively. Based on the subtype-specific sub-pathways, we further identified the sub-pathways related to the prognosis of lung cancer. Similarly, we also identified common and subtype-specific sub-pathways for colorectal cancer (CRC). PathMG is available on the web at https://github.com/dxsbiocc/C-Sub.

## Materials and Methods

### Data and Preprocessing

As described in **Table 1**, the public available somatic mutation profiles, measured by whole-exome sequencing for lung cancer and CRC from six different studies (N. Cancer Genome Atlas, 2012; N. Cancer Genome Atlas Research, 2012; N. Cancer Genome Atlas Research, 2014; Campbell et al., 2016; Giannakis et al., 2016; Ellrott et al., 2018), were downloaded from the cBioPortal (www.cbioportal.org/) database. The mutation profiles of 230 LUAD samples (N. Cancer Genome Atlas Research, 2014) and 178 LSCC samples from Lung-data1 (N. Cancer Genome Atlas Research, 2012) were integrated to identify commonly mutated sub-pathways for lung cancer. The identified common sub-pathways were validated in two independent datasets (Lung-data2 and Lung-data3). For CRC, the mutation profiles of 619 samples from CRC-data1 were used to identify commonly mutated sub-pathways, while two publicly available independent datasets (CRC-data2 and CRC-data3) and one dataset (CRC-data4) measured by our laboratory were used for validation. We measured 13 samples of CRC from five different patients by whole-exome sequencing. For each patient, three specimens were sampled in three different locations. Two specimens with poor DNA quality were excluded from the analysis. The proportion of the tumor epithelial cell was measured by pathological section analysis, ranging from 40 to 100% (**Supplementary Table 1**). This study was approved by the institutional review boards of all participating institutions, and written consent forms were obtained from all participants. All cancer samples were collected from the operating room immediately after surgical resection and were fresh frozen for subsequent DNA extraction. The quantity and quality of extracted DNA was estimated with Qubit 2.0 Fluorometer (Life Technologies, Foster City, CA) by using 2 µl of undiluted DNA solution. The resulting raw whole-exome sequencing files (.fastq) were preprocessed using Trimmomatic (version 0.38) (Bolger et al., 2014), and reads were aligned to the reference genome (GRCh37) using Burrows-Wheeler aligner [BWA; version 0.7.1 (Li and Durbin, 2009)]. Finally, the variant calling was done with variant caller Mutect2 algorithm in GATK4 with high stringency parameters (Cibulskis et al., 2013).

**Table 1:** Description of mutation data used in this study.

Data	Cancer type	Samples	References
Lung-data1	LUAD	230	(N. Cancer Genome Atlas Research, 2014)
	LSCC	178	(N. Cancer Genome Atlas Research, 2012)
Lung-data2	LUAD	562	(Ellrott et al., 2018)
	LSCC	469	
Lung-data3	LUAD	438	(Campbell et al., 2016)
	LSCC	308	
CRC-data1	CRC	619	(Giannakis et al., 2016)
CRC-data2		536	(Ellrott et al., 2018)
CRC-data3		224	(N. Cancer Genome Atlas, 2012)
CRC-data4		13	–

### Kyoto Encyclopedia of Genes and Genomes Pathways

The 239 pathways covering 6,688 unique genes were downloaded from the Kyoto Encyclopedia of Genes and Genomes (KEGG) database (Kanehisa et al., 2010) on October 21, 2018. Here, the human disease pathways were excluded from this study. For each pathway, the interactions between genes were also collected for the following analysis.

### Identify Significantly Mutated Pathways

For a pathway in a given sample, we assume that the pathway is mutated in the sample if at least one gene within the pathway is mutated (Gu et al., 2011; Vandin et al., 2011). Then, for a given pathway *i*, we calculated the number of samples mutated in this pathway for a dataset with *N* samples, denoted as *M_i_*. To test whether the number of mutated samples in the pathway *i* was significantly more than expected by chance, random experiments were performed. For a cancer sample, we calculated the number of mutated genes from its real mutation profile. Simultaneously, to produce a simulated mutation profile for the cancer sample, we randomly selected the same number of genes from the background genes as mutated genes. The total Refseq genes were defined as the background genes. After a random experiment, a random mutation dataset with *N* samples was produced. For a pathway *i*, we could calculate the number of randomly mutated samples after a random experiment, denoted as *R_i_*. The random experiment was repeated *n* (default 1,000) times, which may be adjusted by users. Then, the probability *p_i_*, that the number of randomly mutated samples (*R_i_*) of the pathway *i* is greater than the number of real mutated samples (*M_i_*), is calculated as follows:

(1) $$p_{i}=\frac{\sum_{r=1}^{n}H_{r}}{n}$$

In a random experiment, if *R_i_ > M_i_*, then *H_r_ = 1*; Otherwise, *H_r_ = 0*. The formula was used to calculate the *p* values of all pathways, and the *p* values were adjusted using the Benjamini-Hochberg (BH) method to control the false discovery rate (FDR).

### Identification of Common Sub-Pathways

**Figure 1:**
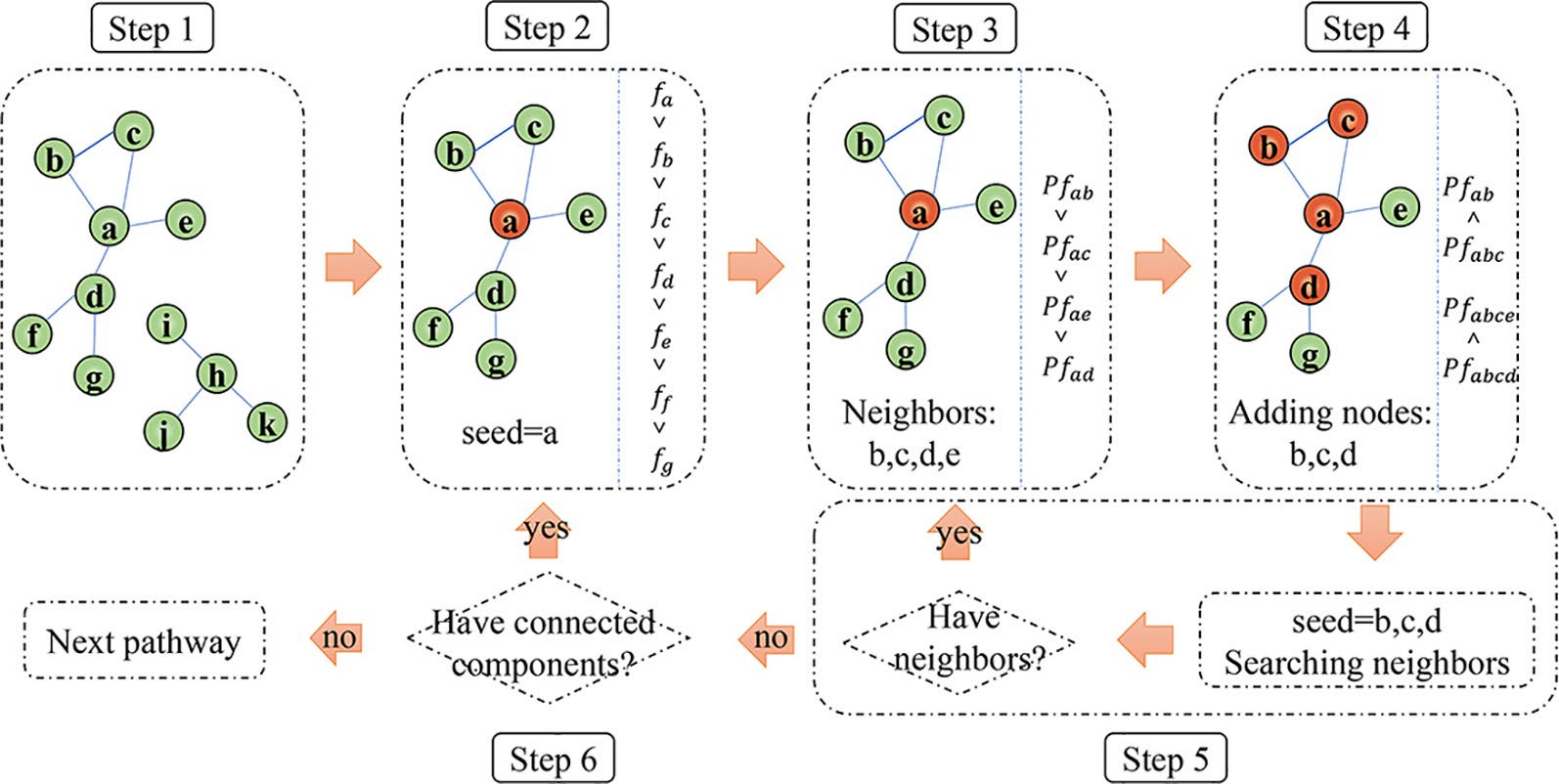
The schematic diagram of the algorithm to identify common mutation sub-pathways. Orange nodes denote genes remained in the sub-pathway.

After identifying the significantly mutated pathways for a cancer, we further extracted common sub-pathways in each of the significant pathways by the greedy search algorithm. Here, the sub-pathways, covering more than 90% (a default parameter) of samples of this cancer, were defined as common sub-pathways. The detailed algorithm to identify common sub-pathways in a given pathway is shown as follows (**Figure 1**).

Step 1: Based on the interactions between genes in a pathway annotated by KEGG database, convert a significant pathway to an undirected graph, and obtain all the connected components on the graph.
Step 2: For a certain connected component, the genes within the connected component are ranked according to the mutation frequency (denoted as *f*) of each gene. Select the gene with the largest *f* value as a seed for an initial sub-pathway.
Step 3: For each of the direct interaction neighbor genes of the seed, calculate the increased coverage (denoted as *Pf*) when the gene is added to the sub-pathway.
Step 4: The direct interaction neighbor genes of the seed will be added to the sub-pathway one by one according to their *Pf* value from high to low. The direct neighbor genes of the seed will be divided into two sets based on whether it increases the coverage of the sub-pathway during the adding process. Then, the genes increasing the coverage are defined as set *GI*; otherwise, defined as set *GN*. The genes in set *GI* are remained in the sub-pathway as new seeds. For each of the genes from set *GN*, its direct neighbor genes will be added one by one according to their *Pf* value from high to low. If at least one neighbor gene could increase the coverage of the sub-pathway, the gene in set *GN* will be remained in the sub-pathway, and its neighbor genes increasing the coverage will be used as new seeds; otherwise, the gene in set *GN* will be excluded. So, we allowed a gene that cannot increase coverage in the process of adding genes.
Step 5: Based on the new seed genes identified in step 4, repeat step 3–4 until the seed genes don’t have direct interaction neighbors in the connected component. If the coverage of the sub-pathway is higher than a predefined parameter (default 90%) and the number of genes in the sub-pathway is at least five (default five), the sub-pathway will be output as a common sub-pathway. The two parameters, the frequency to define common sub-pathway and the minimum number of genes for a sub-pathway, could be adjusted by users.
Step 6: For all the connected components obtained from a significant pathway, repeat steps 2–5 to identify all the common sub-pathways.

### Identification of Subtype-Specific Mutated Pathways and Sub-Pathways

First, the Fisher’s exact test is used to identify the pathways that have significantly different mutation frequencies between two subtypes (subtype *A* and subtype *B*) of a cancer. The significant pathways are defined as subtype-specific mutated pathways. The *p* values are adjusted using the BH method to control the FDR.

After identifying the significantly subtype-specific mutated pathways for a cancer, the sub-pathways, that make the differences of the mutation frequencies between two subtypes as larger as possible, are further extracted in each of the significant pathways using the greedy search algorithm. Similar with the method to identify common sub-pathways, we also integrated the differences of mutation frequencies between subtype *A* and subtype *B* of one cancer and pathway information to identify subtype-specific sub-pathways (**Supplementary Figure 1**). The detailed algorithm to identify subtype-specific sub-pathways in a given pathway is described as follows.

Step 1: Convert a subtype-specific pathway to an undirected graph based on the interactions between genes annotated by KEGG database, and obtain all the connected components on the graph.
Step 2: For a certain connected component, calculate the mutation frequencies of each gene in subtype *A* and subtype *B* of one cancer, respectively, denoted as *f_a_* and *f_b_*. For a given gene, the mutation frequency difference between subtype *A* and subtype *B* is defined as *v*= *f_a_*−*f_b_*. According to the *v* value, the genes with *v*>0 are defined as subtype *A* specific genes (denoted as set *G_a_*) and the genes with *v*>0 are defined as subtype *A* specific genes (denoted as set *G_a_*) and the genes with *v*<0 are defined as subtype *B* specific genes (denoted as set *G_b_*). Then, the two classes of genes (*G_a_* and *G_b_*) are used to identify subtype *A* specific and subtype *B* specific sub-pathways, respectively. Here, we example the process to identify subtype *A* specific sub-pathways to explain the algorithm.
Step 3: Firstly, select the gene with the largest |*v*| value among *G_a_* as a seed for an initial sub-pathway.
Step 4: For each of the direct interaction neighbor genes of the seed in *G_a_*, calculate the increasing of coverage difference (denoted as *Pv*) between subtype *A* and subtype *B* when the gene is added to the sub-pathway.
Step 5: The direct neighbor genes of the seed in *G_a_* will be added to the sub-pathway one by one according to their *Pv* value from high to low. When direct neighbor genes added to the sub-pathway increase the coverage differences, the genes will be remained in the sub-pathway as new seeds. Similarly, PathMG allows for at most one gene that doesn’t increase the coverage difference. Therefore, for a gene that cannot increase coverage difference, its direct neighbor genes will be added one by one according to their *Pv* value from high to low. If at least one neighbor gene could increase the coverage difference of the sub-pathway, the gene will be remained in the sub-pathway and its neighbor genes increased coverage will be used as new seeds; otherwise, the gene will be excluded.
Step 6: Based on the new seeds identified in step 5, repeat step 4–5 until the seeds don’t have direct neighbors among *G_a_* in the connected component. Calculate the *p* values of Fisher’s exact test (*p* < 0.05) for the sub-pathway, and output the subtype *A* specific sub-pathway if its mutation frequency in subtype *A* is higher than subtype *B* with a predefined parameter (default 0.25). Equally, we can identify subtype *B* specific sub-pathways for the connected component.
Step 7: For all the connected components, obtained from a subtype-specific pathway, repeat steps 2–6 to identify all the subtype *A* specific and subtype *B* specific sub-pathways, respectively.

### Sub-Pathways Enriched With Cancer Genes and Drug Target Genes

The cancer genes were downloaded from the Catalogue of Somatic Mutations in Cancer (COSMIC) database, which collected a total of 719 cancer genes (Bamford et al., 2004). We also collected 7,463 target genes for the commonly used drugs for lung cancer therapy, such as carboplatin, cisplatin, and docetaxel, from the Comparative Toxicogenomics Database (http://ctdbase.org/) (Davis et al., 2009). Among the 7,463 target genes, 2,661 genes were included in the KEGG pathways. Simultaneously, 935 target genes for the commonly used drugs for CRC therapy were also downloaded from the Comparative Toxicogenomics Database, among which 527 genes were included in the KEGG pathways.

## Results

### Identify Common Sub-Pathways for Lung Cancer and Colorectal Cancer

Firstly, random experiments were done to identify the significantly mutated pathways. Then, the sub-pathways commonly mutated in at least 90% of patients were identified in each of the significantly mutated pathways. Here, considering the existence of large measurement variation and low quality of tissue samples, we defined the sub-pathways covering more than a predefined parameter (here, default 90%) of patients rather than 100% of patients in a cancer as commonly mutated sub-pathways. The details of the methods were described in *Materials* and *Methods*.

**Figure 2:**
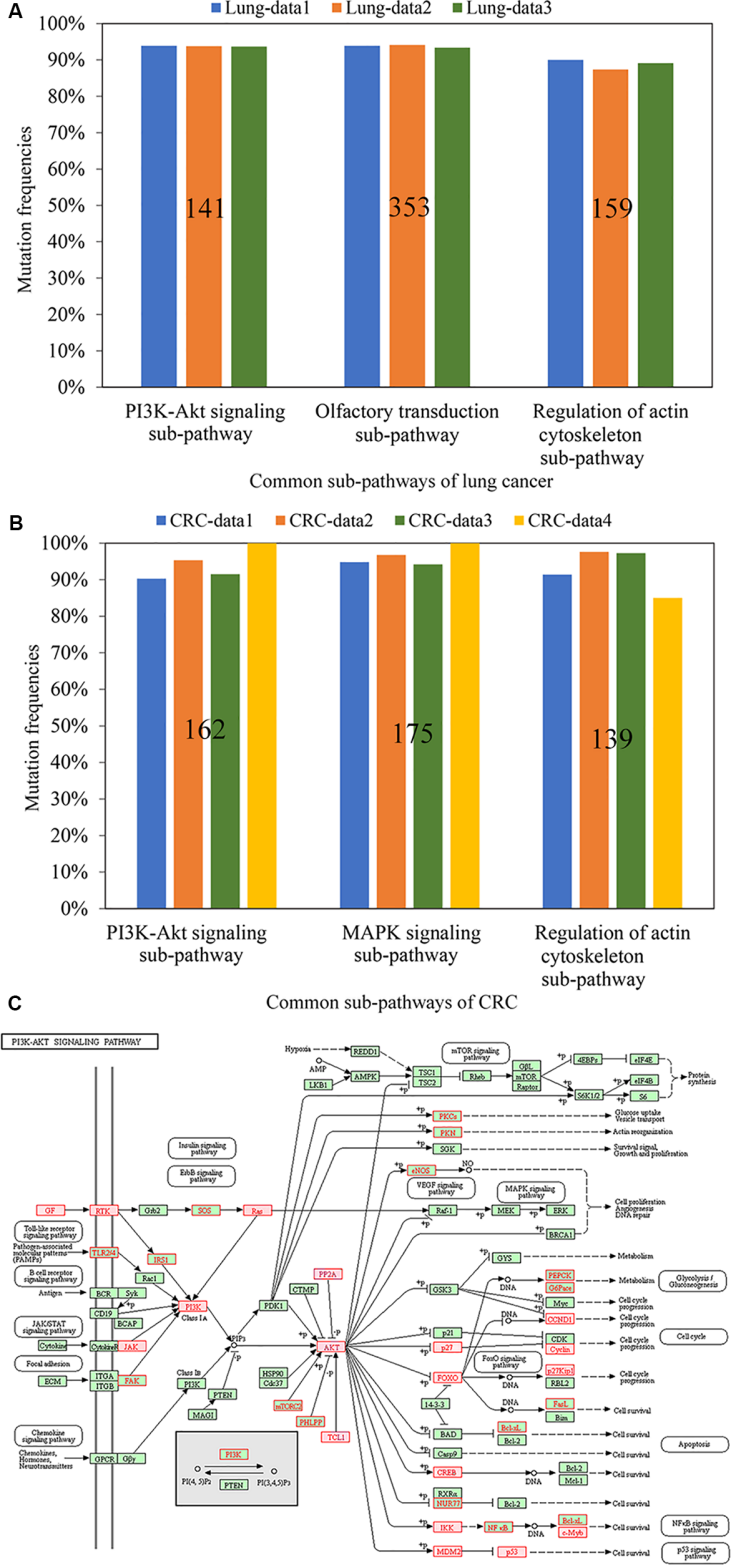
The mutation frequencies of the common sub-pathways across different datasets for lung cancer **(A)** and CRC **(B)**, respectively. The figures on the bars represent the number of genes within the identified sub-pathways. **(C)** The sub-pathway identified from PI3K-Akt signaling pathway. The genes with red font were genes in the sub-pathway and the genes in squares filled with red color were cancer genes.

Using the 408 mutation profiles of Lung-data1, we firstly identified 116 significantly mutated pathways for lung cancer (FDR < 0.05). Then, three sub-pathways, PI3K-Akt signaling sub-pathway, olfactory transduction sub-pathway, and regulation of actin cytoskeleton sub-pathway, were identified as the common sub-pathways of lung cancer (**Figure 2A**). In the two independent validation datasets (Lung-data2 for 1,031 samples and Lung-data3 for 746 samples), two of the three common sub-pathways covered at least 93% samples, whereas the regulation of actin cytoskeleton sub-pathway covered 87 and 89% samples in Lung-data2 and Lung-data3, respectively. The result indicated that the common mutated sub-pathways were highly reproducible in different sets of lung cancer samples, which suggests that the mutation genes within the common sub-pathways could be candidate panels of mutation genes for lung cancer diagnosis. Moreover, except for the sub-pathway of olfactory transduction, two of the three common mutated sub-pathways were significantly enriched with cancer genes documented in the database COSMIC and target genes for the commonly used drugs for lung cancer therapy (**Supplementary Tables 2** and **3**, hypergeometric test, *p* < 0.05). For example, the PI3K-Akt signaling sub-pathway included 141 genes and 43 of them were cancer genes, which was unlikely to happen by chance (**Figure 2C**, hypergeometric test, *p* = 4.28E−29).

Similarly, 125 significantly mutated pathways were identified from the 619 CRC samples from CRC-data1. Among the sub-pathways identified from these significant pathways, three sub-pathways, each of which covered at least 90% of 619 CRC samples, were identified as the common sub-pathways of CRC (**Figure 2B**). Notably, the mutation frequencies of all the three common sub-pathways were higher than 91% in the two independent datasets (CRC-data2 for 536 samples and CRC-data3 for 224 samples, **Figure 2B**). Moreover, all the three reproducible common sub-pathways were significantly enriched with cancer genes documented in the database COSMIC and drug targets for commonly used drugs for colon cancer therapy (**Supplementary Tables 2** and **3**, hypergeometric test, *p* < 0.05).

With whole-exome sequencing, we further measured 13 CRC samples with different proportions of the tumor epithelial cell to validate the three common sub-pathways. For the 13 samples, nine samples were sampled from three patients each with three different tumor locations and the other four samples were sampled from two patients each with two different tumor locations. The results showed that two of the three common sub-pathways covered all the 13 CRC samples. For the remained common sub-pathway of regulation of actin cytoskeleton, it covered 11 of the 13 CRC samples (**Figure 2B**). Overall, these results suggest that these common sub-pathways may be reliable diagnosis marker for CRC even when the proportion of the tumor epithelial cell is as low as 40%.

### Identify Subtype-Specific Sub-Pathways for Lung Cancer and Colorectal Cancer

Based on the mutation profiles of 230 LUAD and 178 LSCC samples from Lung-data1, 43 pathways with significantly different mutation frequencies between LUAD and LSCC were identified using Fisher’s exact test (FDR < 0.05). Here, we developed an algorithm to identify subtype-specific sub-pathways with at least 0.25 mutation frequency difference between LUAD and LSCC (*p* < 0.05).

**Figure 3:**
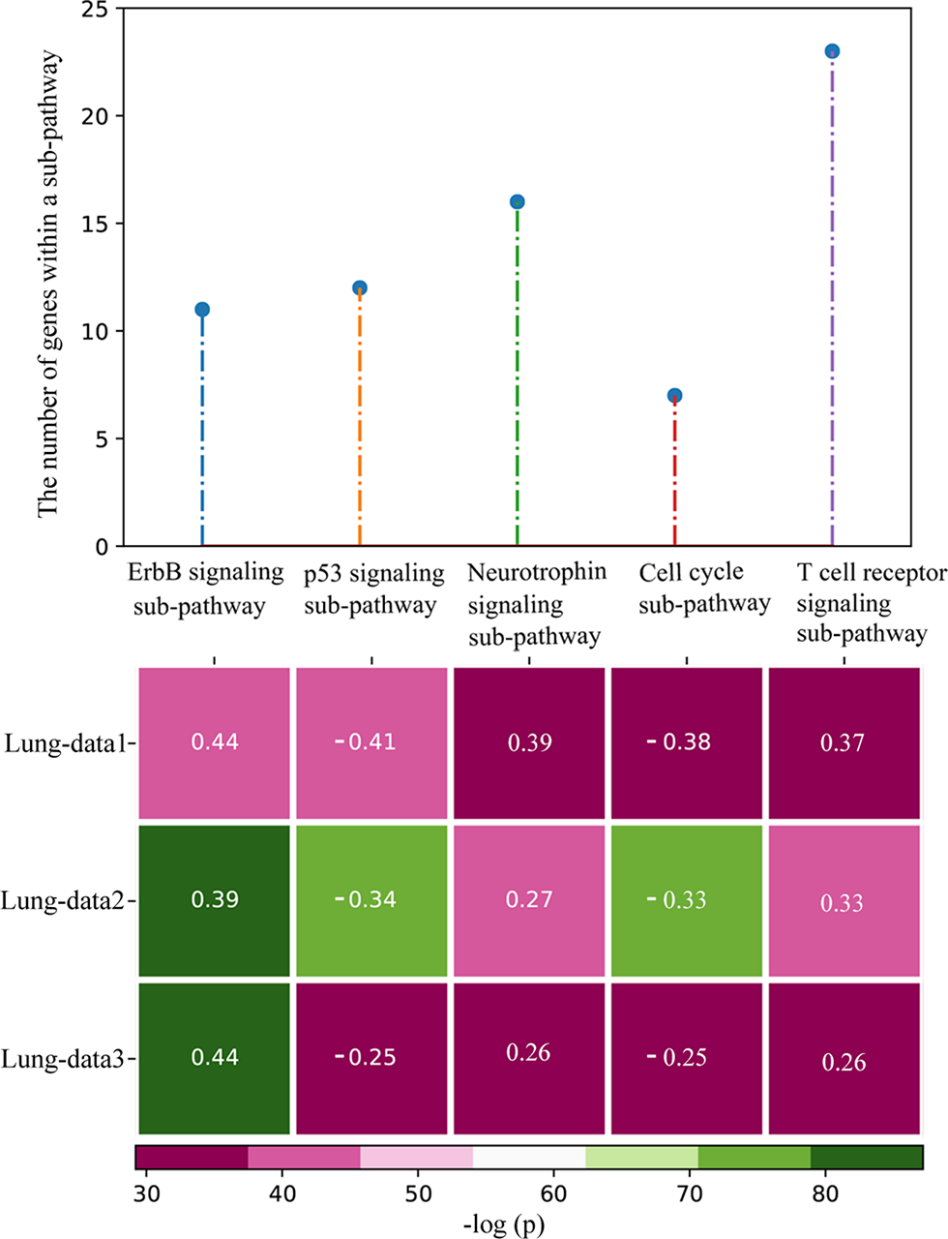
The top five most significant subtype-specific sub-pathways with the largest differences of mutation frequencies. The heatmap shows the *p* values of the sub-pathways calculated by Fisher’s exact test, and the figures on the heatmap represent the mutation frequency differences between lung adenocarcinoma (LUAD) and lung squamous cell carcinoma (LSCC). The mutation frequency difference was calculated as the mutation frequency of the sub-pathway in LUAD minus the mutation frequency of the sub-pathway in LSCC. When the figure on the heatmap was positive (negative), the sub-pathway was LUAD-specific (LSCC-specific) sub-pathway.

Based on the 43 subtype-specific pathways, a total of 13 subtype-specific sub-pathways with at least 0.25 mutation frequency difference between the 230 LUAD and 178 LSCC samples were identified in Lung-data1 (*p* < 0.05), including 6 LUAD-specific sub-pathways and 7 LSCC-specific sub-pathways. In the two independent Lung-data2 and Lung-data3 datasets, all the six LUAD-specific sub-pathways were validated as LUAD-specific sub-pathways. For the seven LSCC-specific sub-pathways, six were validated as LSCC-specific sub-pathways. Only one sub-pathway, inositol phosphate metabolism, had *p* value less than 0.05 in both two validation datasets, but its mutation frequency difference was 0.18 and 0.12 in Lung-data2 and Lung-data3, respectively (**Supplementary Table 4**). Notably, all the top five sub-pathways with the largest differences of mutation frequencies between LUAD and LSCC in Lung-data1 were reproducible in both the two independent datasets (**Figure 3**).

**Figure 4:**
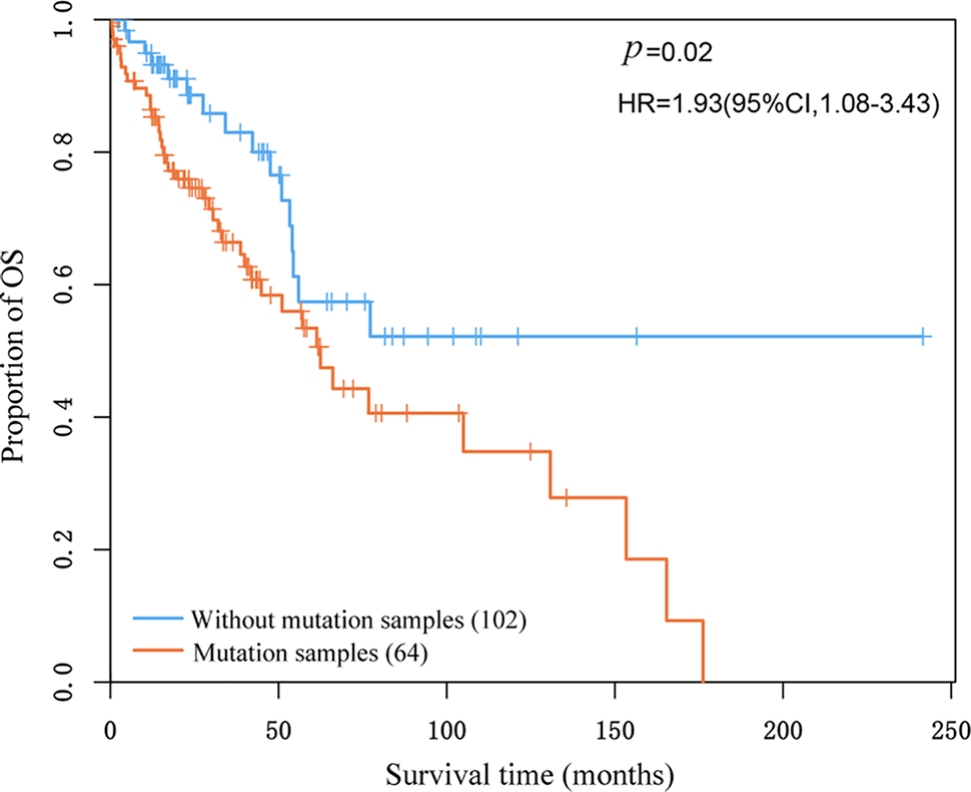
Kaplan–Meier estimates of overall survival according to whether lung squamous cell carcinoma-specific sub-pathway of cell cycle mutated in the patients.

Based on the knowledge that LSCC patients suffered poorer prognoses than LUAD patients (Gyorffy et al., 2013), we performed survival analysis using the overall survival data of the 87 LUAD and 79 LSCC samples from Lung-data1. These patients were at the stage I and treated with complete surgical resection to exclude the effects of stage and chemotherapy on prognosis. We evaluated whether the patients with and without mutation of a sub-pathway were significantly different in overall survival (OS) time. Finally, five of the 12 reproducible subtype-specific sub-pathways were found to be associated with OS (the univariate Cox proportional-hazards regression model, *p* < 0.05) (**Supplementary Table 5**). Among the five top sub-pathways, three sub-pathways, p53 signaling pathway, T cell receptor signaling pathway, and cell cycle, were related to the OS of lung cancer. For example, a LSCC-specific sub-pathway of cell cycle, including seven genes, was mutated in 102 of 166 patients, which had significantly poorer overall survival than the other 64 patients without the mutation of this sub-pathway (log-rank *p* = 0.02, **Figure 4**).

Similarly, 221 subtype-specific pathways were identified for CRC using the mutation profiles of 166 LCC and 315 RCC samples from CRC-data1 (Fisher’s exact test, FDR < 0.05). Based on the subtype-specific pathways, 42 subtype-specific sub-pathways were further identified with mutation frequency difference higher than 0.25 (*p* < 0.05). All the 42 sub-pathways were RCC-specific, which further validated the report that RCC was hyper-mutational (N. Cancer Genome Atlas, 2012). Because only CRC-data3 had the information of tumor location in the validation datasets, we then validated the 42 RCC-specific sub-pathways in CRC-data3. Among the 42 RCC-specific sub-pathways, 34 sub-pathways were validated in CRC-data3 (**Supplementary Table 4**). For the remained eight sub-pathways, only two sub-pathways had *p* ≥ 0.05 and the other six sub-pathways had *p* < 0.05 with mutation frequency differences ranging from 0.17 to 0.24 in CRC-data3. Moreover, 39 of the 42 RCC-specific sub-pathways were enriched with cancer genes, and 32 of the 42 RCC-specific sub-pathways were enriched with target genes for the commonly used CRC therapy drugs (**Supplementary Table 6**, hypergeometric test, *p* < 0.05).

## Discussion

In this study, we developed an empirical algorithm, named PathMG, to identify commonly mutated sub-pathways for a specific cancer. For lung cancer, two of three common sub-pathways were identified from the PI3K-Akt signaling pathway and the regulation of actin cytoskeleton pathway, which were known cancer hallmarks (Hanahan and Weinberg, 2000; Hanahan and Weinberg, 2011). Both the two sub-pathways were enriched with cancer genes and cancer drug targets. Another common sub-pathway extracted from olfactory transduction pathway covered more than 90% samples of each dataset for lung cancer. It has been reported that olfactory transduction pathway can affect apoptosis of lung cancer cells (Lu et al., 2013), which may be new hallmark of lung cancer. Similarly, we also identified three common sub-pathways for CRC. Among them, two common sub-pathways were also identified from PI3K-Akt signaling pathway and Regulation of actin cytoskeleton pathway. It suggests that the two pathways may be hallmark for pan-cancer. Due to the high reproducibility of common sub-pathways, they may be reliable cancer diagnosis markers. Especially, the common sub-pathways identified in CRC were reproducible even when the proportion of the tumor epithelial cell was as low as 40%. Because the mutation profiles of circulating tumor DNA (ctDNA) were lacking, we only applied the algorithm to identify common sub-pathways using mutation profiles of tissues. However, the application of the algorithm is not restricted to tissues, it can also be used to analyze mutation profiles of ctDNA.

Simultaneously, PathMG can provide definite subtype-specific sub-pathways for a cancer with two known subtypes, which may give a novel way to identify subtype diagnosis signatures. Here, we identified six reproducible LUAD-specific sub-pathways and six reproducible LSCC-specific sub-pathways for lung cancer. Most of these sub-pathways were enriched with cancer genes and target genes for the commonly used lung cancer drugs (**Supplementary Table 6**). Similarly, we also identified 42 subtype-specific sub-pathways for CRC. All the sub-pathways were RCC-specific, which further validated that RCC had higher mutation rate than LCC (N. Cancer Genome Atlas, 2012).

Here, the default coverage to identify common sub-pathways was defined as 90% which can be adjusted by users. This parameter will affect the discovery of the number of common sub-pathways for a particular cancer. When the parameter was defined as 85%, six common sub-pathways were identified in Lung-data1. In the two independent validation datasets (Lung-data2 and Lung-data3), five of the six common sub-pathways covered at least 86% samples, whereas the remained sub-pathway of phospholipase D signaling pathway covered 84% samples in both the two validation datasets (**Supplementary Table 7**). Similarly, seven reproducible common sub-pathways were obtained for CRC (**Supplementary Table 7**). The results indicated that the common sub-pathways identified in different coverages (90 or 85%) were highly reproducible. For subtype-specific sub-pathway analysis, we considered the mutation frequency difference of a sub-pathway between two subtypes of a cancer as a parameter, and the default value was defined as 0.25. As expected, the larger the parameter, the more likely the discovered sub-pathways to be reproducible in independent validated datasets. For example, the top 10 sub-pathways in Lung-data1 were ranked within the top 12 sub-pathways in the two independent validation datasets (**Supplementary Table 8**).

Besides, because all the mutation profiles analyzed in this study were detected by whole-exome sequencing, the total exon length of a gene will affect the mutation frequency of a gene theoretically. To analyze the effect, we studied the distribution of mutation counts among genes with different total exon lengths. The result showed that the mutated genes with total exon lengths shorter than 18,000 bp accounted for about 94% mutated genes (**Supplementary Figure 2**). The average mutation counts of these genes ranged from 4.6 to 10.3, indicating that the mutation counts didn’t vary widely for most genes. Then, we didn’t consider this factor. One possible way of addressing this limitation is to use the algorithm, called PathScan, to identify significant pathways which considered the effect of gene length (Wendl et al., 2011). Then, we can identify common sub-pathways based on the significant pathways identified by PathScan. The number of random experiments was also a limitation of PathMG. To assure the power of the algorithm, the experiment was repeated 1,000 times which could be adjusted by users. In this study, we only analyzed KEGG pathways to interpret the algorithm of PathMG, which will limit the number of identified common and subtype-specific sub-pathways. When using PathMG to identify diagnostic markers for a cancer, we had better integrate more pathways from different databases to obtain the optimal sub-pathway marker.

In summary, PathMG can be used to identify common and subtype-specific sub-pathways for a particular cancer, which may help users to prioritize panels of mutations at the sub-pathway level to aid cancer diagnosis and sub-pathway targeted treatment.

## Data Availability Statement

The raw data supporting the conclusions of this manuscript will be made available by the authors, without undue reservation, to any qualified researcher.

## Ethics Statement

The studies involving human participants were reviewed and approved by Fujian medical university biomedical research ethics committee. The patients/participants provided their written informed consent to participate in this study. Written informed consent was obtained from the individual(s) for the publication of any potentially identifiable images or data included in this article.

## Author Contributions

ZG, HY, and XD conceived and designed the overall study. HY, XD, HC, JC, JH, QG, ML, JXie and JXia analyzed the data. HY, YG and ZG wrote the manuscript. HY, XD and HC revised the manuscript. All authors read and approved the final manuscript.

## Funding

This work was supported by the National Natural Science Foundation of China [grant numbers. 61801118]. The education research project for young and middle-aged teachers in Fujian province [grant number. JAT170214] and the Fujian natural science foundation [grant number. 2019J01678].

## Conflict of Interest

The authors declare that the research was conducted in the absence of any commercial or financial relationships that could be construed as a potential conflict of interest.
