# Supplementary material for: Identification of Common and Subtype-Specific Mutated Sub-Pathways for a Cancer
Source: Front Genet. 2019 Nov 28;10:1228. doi: 10.3389/fgene.2019.01228 (PMC6892778; doi:10.3389/fgene.2019.01228)
Supplement: Supplementary file 1 [file DataSheet_1.zip › Figures S1, S2.doc]

**Supplementary Figures**

**Supplementary Figure 1.** The schematic diagram of the algorithm to identify subtype-specific mutation sub-pathways. Orange nodes denote genes remained in the sub-pathway.

**Supplementary Figure 2.** Impact of the exon length on the mutation frequencies of genes. Blue bars represent the average mutation counts of genes, and red line represents the number of gene counts with different exon lengths.
