# Supplementary material for: Identification of Common and Subtype-Specific Mutated Sub-Pathways for a Cancer
Source: Front Genet. 2019 Nov 28;10:1228. doi: 10.3389/fgene.2019.01228 (PMC6892778; doi:10.3389/fgene.2019.01228)
Supplement: Supplementary file 1 [file DataSheet_1.zip › Tables S1, S2, S3, S5, S7.DOC]

**Supplementary tables**

**Supplementary Table 1.** The proportions of the tumor epithelial cell for CRC tissues of each patient sampled from three different locations.

| Sample | Proportion 1 | Proportion 2 | Proportion 3 |
| --- | --- | --- | --- |
| S1 | 70% | 40% | - |
| S2 | 40% | 100% | 100% |
| S3 | 50% | 90% | 90% |
| S4 | 60% | 100% | 100% |
| S5 | 100% | 100% | - |

**Supplementary Table 2.** The cancer gene enrichment analysis of common sub-pathways for lung cancer and CRC using hypergeometric test.

| Sub-pathway | Number of genes in a sub-pathway | Number of cancer genes | *P* value |
| --- | --- | --- | --- |
| **Lung cancer** |  |  |  |
| PI3K-Akt signaling sub-pathway | 141 | 43 | 3.24E-11 |
| Olfactory transduction sub-pathway | 353 | 0 | 1 |
| Regulation of actin cytoskeleton sub-pathway | 159 | 28 | 4.32E-11 |
| **CRC** |  |  |  |
| MAPK signaling sub-pathway | 175 | 43 | 1.11E-11 |
| Regulation of actin cytoskeleton sub-pathway | 139 | 30 | 4.15E-11 |
| PI3K-Akt signaling sub-pathway | 162 | 49 | 1.35E-12 |

**Supplementary Table 3.** The drug target gene enrichment analysis of common sub-pathways for lung cancer and CRC using hypergeometric test.

| Sub-pathway | Number of genes in a sub-pathway | Number of drug target genes | *P* value |
| --- | --- | --- | --- |
| **Lung cancer** |  |  |  |
| PI3K-Akt signaling sub-pathway | 141 | 84 | 9.45E-11 |
| Olfactory transduction sub-pathway | 353 | 1 | 1 |
| Regulation of actin cytoskeleton sub-pathway | 159 | 80 | 3.75E-06 |
| **CRC** |  |  |  |
| MAPK signaling sub-pathway | 175 | 105 | 1.00E-16 |
| Regulation of actin cytoskeleton sub-pathway | 139 | 78 | 1.36E-08 |
| PI3K-Akt signaling sub-pathway | 162 | 97 | 1.00E-16 |

**Supplementary Table 5.** The overall survival time related sub-pathways in lung cancer.

| Sub-pathways | Type | Number of genes | P value | HR |
| --- | --- | --- | --- | --- |
| Apoptosis subpathway | LUAD-specific | 9 | 0.02 | 0.35 |
| p53 signaling pathway sub-pathway | LSCC-specific | 12 | 0.02 | 1.99 |
| Cell cycle sub-pathway | LSCC-specific | 7 | 0.03 | 1.93 |
| Thyroid hormone signaling sub-pathway | LSCC-specific | 4 | 0.04 | 1.80 |
| T cell receptor signaling sub-pathway | LUAD-specific | 26 | 0.04 | 0.54 |

.

**Supplementary Table 7.** Sub-pathways covering at least 85% of samples in Lung-data1 and CRC-data1.

| Sub-pathway | Number of genes | Mutation frequencies | | | | | |
| --- | --- | --- | --- | --- | --- | --- | --- |
| Lung cancer |  | | Lung-data1 | Lung-data2 | | Lung-data3 | |
| PI3K-Akt signaling sub-pathway | 141 | | 0.939 | 0.938 | | 0.937 | |
| Olfactory transduction sub-pathway | 353 | | 0.939 | 0.941 | | 0.934 | |
| Regulation of actin cytoskeleton sub-pathway | 159 | | 0.900 | 0.874 | | 0.891 | |
| Neurotrophin signaling sub-pathway | 72 | | 0.892 | 0.880 | | 0.882 | |
| cAMP signaling sub-pathway | 124 | | 0.890 | 0.865 | | 0.883 | |
| Phospholipase D signaling sub-pathway | 83 | | 0.863 | 0.848 | | 0.843 | |
| CRC |  | | CRC-data1 | | CRC-data2 | | CRC-data3 |
| MAPK signaling pathway sub-pathway | 175 | | 0.948 | | 0.968 | | 0.942 |
| Regulation of actin cytoskeleton sub-pathway | 139 | | 0.914 | | 0.976 | | 0.973 |
| PI3K-Akt signaling pathway sub-pathway | 162 | | 0.903 | | 0.953 | | 0.915 |
| Neurotrophin signaling sub-pathway | 69 | | 0.885 | | 0.942 | | 0.902 |
| Wnt signaling sub-pathway | 94 | | 0.880 | | 0.976 | | 0.969 |
| cAMP signaling sub-pathway | 156 | | 0.872 | | 0.888 | | 0.746 |
| Cellular senescence sub-pathway | 62 | | 0.859 | | 0.937 | | 0.884 |
